# Intraoperative Identification and Mosaicplasty in a Case of Femur Subchondral Osteoid Osteoma

**DOI:** 10.7759/cureus.47393

**Published:** 2023-10-20

**Authors:** Vishal S Patil, Rishabh Aggarwal, Archit Gupta, Sushant Kumar, Vinod Nair

**Affiliations:** 1 Department of Orthopaedics, Dr. D. Y. Patil Medical College, Hospital and Research Centre, Pune, IND

**Keywords:** needle pricking method, subchondral, arthrotomy, mosaicplasty, osteoid osteoma

## Abstract

Osteoid osteomas (OOs) are non-malignant primary bone abnormalities marked by a central nidus surrounded by reactive sclerosis. They typically manifest as aggravated nocturnal pain that responds to non-steroidal anti-inflammatory drugs (NSAIDs). These growths are most frequently found within the intracortical bone and the diaphysis of elongated bones. Within the realm of uncommon conditions, intra-articular OOs (IAOOs) exhibit distinctive presentations, often leading to postponed or inaccurate diagnoses. We present a patient with OO at the distal femur, accessible through the knee joint, which was intraoperatively identified and localized using a *needle pricking technique* and treated by arthrotomy and mosaicplasty.

## Introduction

Osteoid osteoma (OO) is a non-malignant, osteoblastic, and painful tumor-like lesion. It ranks as the third most prevalent benign bone lesion, with intra-articular cases comprising around 5% to 13% of occurrences [1-3]. Within the category of intra-articular OOs (IAOOs), sometimes referred to as "juxta-articular OOs," these are characterized by their development within synovial cavities, specifically in subsynovial or subchondral locations. Subchondral IAOOs stand out due to their proximity to and engagement with articular cartilage [2]. Unlike their extra-articular counterparts located in the diaphysis, IAOOs often exhibit atypical radiographic and advanced imaging manifestations, contributing to delays in diagnosis, taking over two years on average, in contrast to 8.5 months for extra-articular cases [1,2,4,5].

Upon recognition, intervention is generally required for IAOOs. Minimally invasive approaches aided by imaging, such as CT-guided radiofrequency ablation (CT-RFA), present potential strategies. However, these methods carry theoretical risks of affecting cartilage and lack comprehensive exploration for subchondral IAOO treatment [6,7]. Surgical extraction can be done through open or arthroscopic procedures, yet preserving articular cartilage proves challenging, particularly in weight-bearing regions [3,8,9].

Presented here is a case of an IAOO situated in the distal femur at a subchondral location. This case was identified and localized intraoperatively using a needle pricking technique and treated with arthrotomy and mosaicplasty.

## Case presentation

Clinical history

A 36-year-old male reported to the OPD and complained of a 4-year history of atraumatic pain in the left knee which was undiagnosed on three previous MRIs.

The pain emerged gradually, with a subtle onset and mild intensity at first, but over the course of several months, it steadily worsened. This persistent discomfort was present throughout both daytime and nighttime, and while its severity was somewhat relieved by non-steroidal anti-inflammatory drugs (NSAIDs), it persisted despite treatment.

Upon physical examination, there was noticeable but mild swelling and tenderness at the medial femoral condyle. No joint effusion or quadriceps atrophy was observed. The range of motion of the knee extended from 0 to 110 degrees, showing symmetrical symmetry with the opposite knee. Evaluation of the patellofemoral joint revealed no abnormalities.

**Figure 1:**
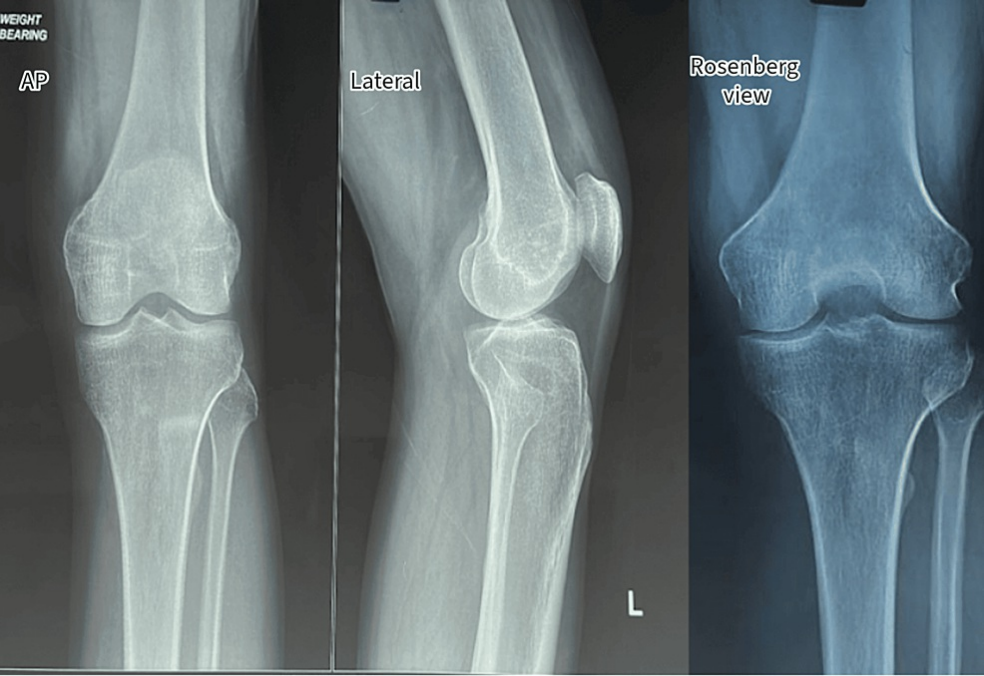
Preoperative plain radiograph

**Figure 2:**
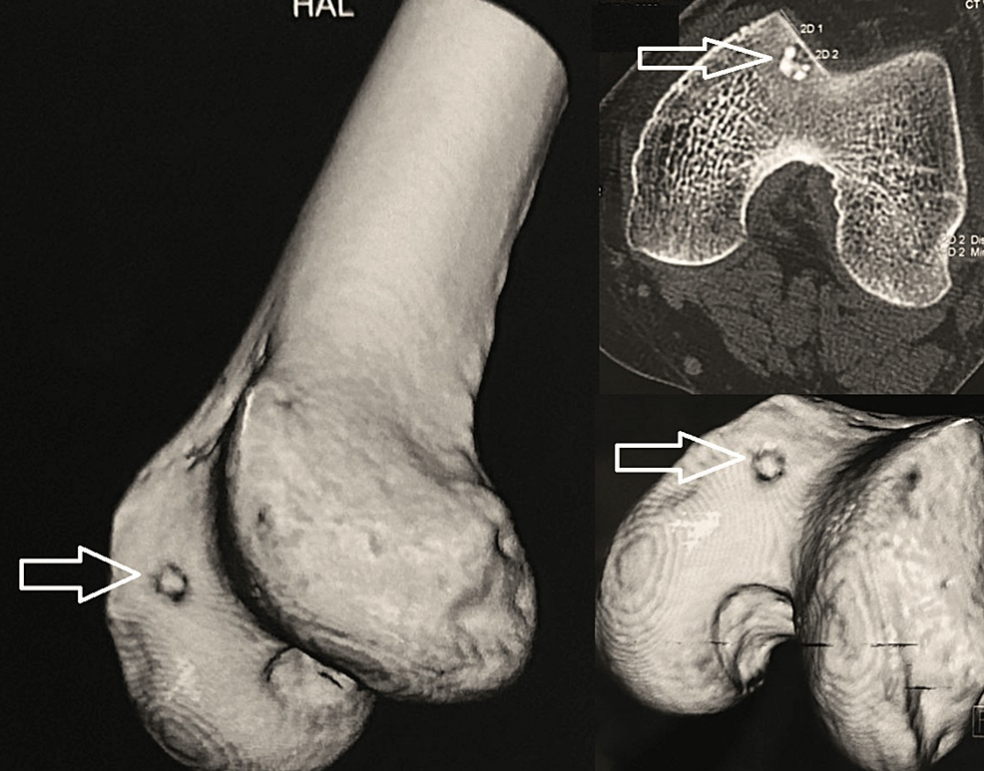
CT scan showing left knee joint Arrows showing the lesion

**Figure 3:**
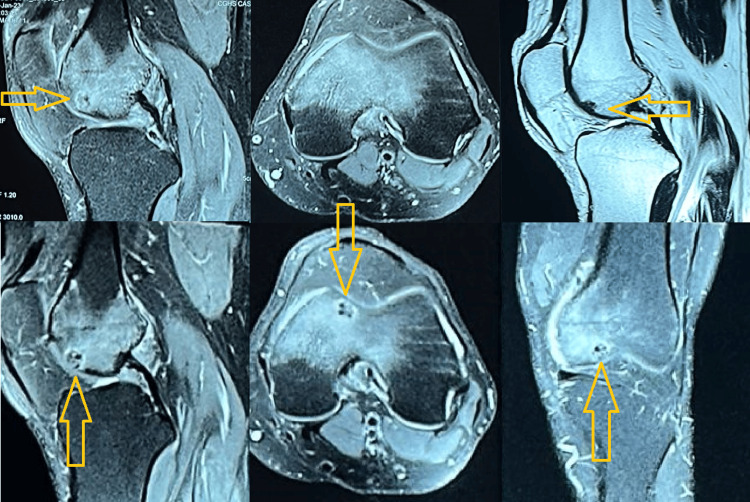
MRI showing the left knee joint Arrows showing the lesion

A knee X-ray was conducted, yielding unremarkable results (Figure 1). Subsequently, a CT scan (Figure 2) and an MRI were performed (Figure 3). These imaging techniques revealed a distinct, well-defined circular lesion measuring 8x6 mm located in the front part of the medial femoral condyle. Furthermore, the MRI indicated areas of uneven bone marrow swelling within the medial femoral condyle, along with a noticeable accumulation of fluid in the knee joint. The differential diagnosis encompassed subchondral cysts and OOs.

**Figure 4:**
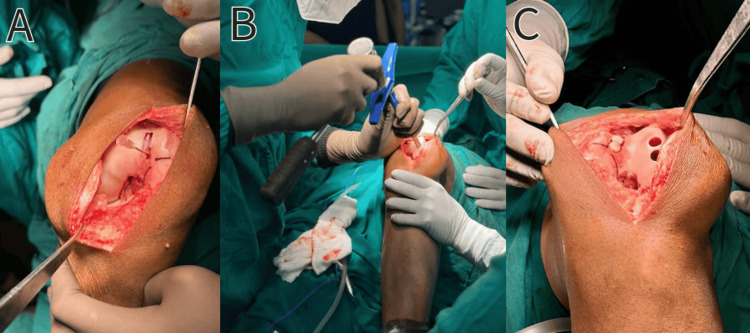
Intraoperative pictures (A) Intraoperative identification of the lesion using a needle pricking technique. (B) Lesion taken out using a harvester. (C) Graft harvested from the lateral femoral condyle and inserted into the medial condyle

We had planned an open arthrotomy of the knee joint and excision of the tumor with reconstruction by mosaicplasty. The individual was positioned in a supine stance with spinal anesthesia administered. A medial parapatellar incision was made, and the patella was flipped laterally, revealing the lesion located over the distal medial femoral condyle. The cartilage over the trochlea and medial femoral condyle was normal. On C-Arm, the lesion was not identified. The preoperative CT scans had pinpointed a lesion measuring between 6 and 8 mm in diameter and around 1 mm below the subchondral bone. As a very thin bone wall was present between the normal cartilage and the tumor, we decided to identify the tumor by our needle pricking technique with an 18G needle for precise intraoperative identification, localization, and depth assessment (as shown in Figure 4A). We measured the preoperative distances of the tumor from three bony edges, and intraoperatively, we marked the same measurements with a marker pen (as shown in Figure 4A). After identifying the small area between the marked measurements, an 18G needle was used to prick the normal cartilage. At the site of the tumor, the needle was pierced deep around 1 cm, and repeating the same, we localized the whole tumor. The cartilage over the tumor was removed, and curettage of the tumor was done. After curettage, we had a defect of 14x8 mm. Utilizing a mosaicplasty kit (8 mm Arthrex), we prepared two recipient wells at the site of the excised tumor. Two osteochondral grafts of size 8 mm were harvested from the non-weight-bearing part of the lateral femoral condyle and put in the prepared recipient site. With mosaicplasty, we could reconstruct the cartilage defect (as depicted in Figure 4B-4C).

**Figure 5:**
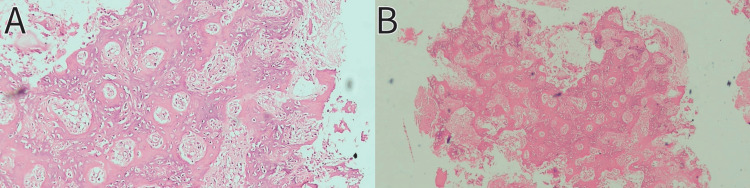
Histopathological slides of the intraoperative sample confirmed the diagnosis to be OO (A) Magnification: 400X. (B) Magnification: 100X. Stain - hematoxylin and eosin

**Figure 6:**
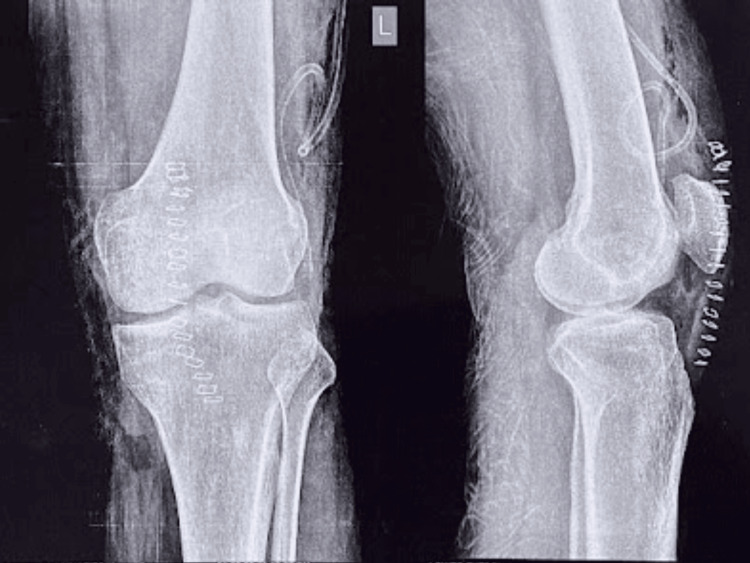
Postoperative plain radiograph of the left knee joint

Intraoperative samples were sent for histopathological examination which confirmed the diagnosis to be OO (Figure 5), and a postoperative X-ray the of left knee joint was done (Figure 6).

Postoperative rehabilitation

The patient was kept partial weight bearing with a long knee brace for six weeks. The knee range of motion was restricted up to 60 degrees for three weeks and later improved to complete flexion by the end of eight weeks, and full weight bearing was allowed at the end of eight weeks. Further quadriceps strengthening and sports activities were started at around six months. The patient was comfortable with a full range of motion and joined his job in the security forces at the end of eight months.

## Discussion

IAOO is commonly observed in the hip joint, with a primary prevalence in the upper part of the femur. Nonetheless, occurrences of IAOO have been noted in different joints such as the knee, ankle, elbow, hand, subtalar, and metatarsophalangeal joints [1,2,10]. Regular X-rays often fail to promptly detect IAOO since the distinctive hardened border is usually absent in most cases [2,5].

The plan can be to make a patient-specific 3D printed jig to localize the tumor unavailable at our institute. However, MRI plays a vital role in distinguishing osteochondral defects, subchondral cysts, infections, or stress fractures, but it does not display IAOO early as profound marrow edema, effusion, and no sclerosis around the lesion in the early stage, making it difficult to differentiate with infection [6,11-13]. CT scans are the favored diagnostic imaging technique, especially when using 3 mm thick sections or smaller to precisely identify small niduses [6,12].

While NSAIDs are the mainstay for OO treatment, addressing IAOOs is more complex due to its connection with synovitis-associated joint complications, resulting in heightened morbidity [4,14]. CT-RFA is the recognized interventional treatment for classic, extra-articular OOs, boasting success rates exceeding 90% [15]. However, applying this technique to IAOOs raises concerns about potential chondrocyte demise attributed to thermal injury. Previous studies involving cadavers and animals have indicated a 1 cm area of damage resulting from CT-RFA [16-18]. Utilizing radiofrequency ablation poses a potential risk of cartilage damage above the lesion and possibly insufficiently addressing pathologically compromised cartilage. Conversely, while straightforward excision may offer pain relief, it fails to adequately address the subsequent loss of articular cartilage. Nevertheless, various studies indicate the use of CT-RFA as a treatment for IAOO, demonstrating results similar to those seen in cases of diaphyseal OO [7,15,19,20]. It's worth noting that the ablation procedure typically avoids a transarticular approach, especially in hip IAOO cases, with only a limited number of instances involving direct subchondral sites. To address this uncommon scenario, we propose an alternative treatment approach, involving the needle pricking technique described in the procedure with arthrotomy followed by mosaicplasty. However, it can be done only if the tumor wall is 1-2 mm thin and cannot be performed in deeper IAOOs. This innovative method has demonstrated positive outcomes, including effective pain alleviation and the restoration of the articular surface.

The primary surgical intervention for IAOOs has commonly involved straightforward excision, employing both open and arthroscopic methods [3,8,9,11]. Various accounts of distal femoral lesions underscore favorable pain alleviation and functional outcomes achieved through excision. However, it's important to note that these accounts exclusively pertain to subsynovial lesions that do not induce damage to the weight-bearing zone of the cartilage [3,8,9]. Hence, in specific patient cases, osteochondral grafting presents various benefits. This approach addresses a range of potential diagnoses, including osteochondral defects, allowing appropriate treatment for either condition. After complete excision, a definitive diagnosis is more feasible due to the sufficient biopsy sample, as confirmed pathology results were only achieved in 73% of a significant series of OOs [15]. Osteochondral grafting represents a well-established approach for addressing cartilage defects in the distal femur, thus presenting a rational therapeutic choice for this specific site. While the patient in our case had reached skeletal maturity, it is conceivable that this technique could be adapted for pediatric patients with adequate epiphyseal size, allowing for resection without influencing the growth plate.

## Conclusions

We introduce a case involving a subchondral IAOO located within the distal femur. In such cases, intraoperative identification of the tumor is difficult because of the normal overlying cartilage. For the localization of the tumor, we used our needle pricking technique with preoperative CT planning, which is easy and cheaper than making a 3D-printed patient-specific jig. We advocate using this technique in tumors with a very thin wall of subchondral bone between the tumor and cartilage. This ingenious method exemplified positive outcomes, effective pain alleviation, and the restoration of the articular surface.
